# Estimation of the Path-Loss Exponent by Bayesian Filtering Method

**DOI:** 10.3390/s21061934

**Published:** 2021-03-10

**Authors:** Piotr Wojcicki, Tomasz Zientarski, Malgorzata Charytanowicz, Edyta Lukasik

**Affiliations:** Department of Computer Science, Faculty of Electrical Engineering and Computer Science, Lublin University of Technology, Nadbystrzycka 38D, 20-618 Lublin, Poland; t.zientarski@pollub.pl (T.Z.); m.charytanowicz@pollub.pl (M.C.); e.lukasik@pollub.pl (E.L.)

**Keywords:** path loss exponent, particle filter, Bayesian filtering, received signal strength, WSN

## Abstract

Regarding wireless sensor network parameter estimation of the propagation model is a most important issue. Variations of the received signal strength indicator (RSSI) parameter are a fundamental problem of a system based on signal strength. In the present paper, we propose an algorithm based on Bayesian filtering techniques for estimating the path-loss exponent of the log-normal shadowing propagation model for outdoor RSSI measurements. Furthermore, in a series of experiments, we will demonstrate the usefulness of the particle filter for estimating the RSSI data. The stability of this algorithm and the differences in determined path-loss exponent for both method were also analysed. The proposed method of dynamic estimation results in significant improvements of the accuracy of RSSI values when compared with the experimental measurements. It should be emphasised that the path-loss exponent mainly depends on the RSSI data. Our results also indicate that increasing the number of inserted particles does not significantly raise the quality of the estimated parameters.

## 1. Introduction

The ubiquitous wireless technology, in addition to providing easy connection to the network, fulfils many other functions in everyday life. The radio signal used in protocols ZigBee, WiFi, or Bluetooth, apart from the basic functionality of data transmission, may be used for another purpose. The received signal indirectly provides a lot of information and can therefore be implemented in many ways. One of the primary uses are attempts to locate an object (device) on the basis of received signal strength indicator (RSSI). Many papers describe the use of RSSI for indoor location [1,2,3,4,5,6,7] and outdoor location [8,9,10,11,12,13]. The main reason for these applications is the wide availability and affordability of devices using the received signal strength indicator. Additional advantages of these devices are low-level energy consumption and quick access to the RSSI [2].

Depending on the signal strength between the transmitter (e.g., router) and receiver (e.g., smartphone), it is possible to estimate the distance between them. A complex floor plan with many obstacles may cause inaccurate measurement of distance. Using several transmitters permanently placed in known locations allows a more precise determination of the area in which the receiver is located. The use of trilateration techniques in this case may not give satisfactory results due to fluctuations of the signal caused by multipath propagation and shadowing effect. Indoors these techniques are more accurate than those based on GPS [1,3,8].

In wireless sensor networks (WSNs), a set of sensors that have been put into place over a large area for monitoring, e.g., environmental properties, requires correct data transmission between network nodes. In order to determine the position of an unknown node in the network, the distance between it and the known nodes must first be determined. This can be achieved using the RSSI. Communication is the most energy-consuming process in all operations of a sensor network. Moreover, the method based on RSSI measurements also does not introduce communication overhead, which is definitely an advantageous feature in the case of WSN nodes, which typically have limited power [14,15,16,17].

Due to the characteristics of wireless transmissions, a signal received from one place at several intervals has variable strength [1,3]. The process of estimating the distance between a transmitter and a receiver requires an appropriate model. Even such a simple concept of solving this problem can be really difficult at times. This is due to two main factors. The first one is directly related to the specificity of the RSSI signal. Here, the measured RSSI values strongly depend on the conditions under which these measurements are made. The second factor is the difficulty of determining an appropriate optimal path-loss model under given conditions [18,19].

In the context of WSNs, three models based on RSSI signal propagation attract the most attention. They are: the free-space model, the 2-ray ground model, and the log-normal shadow model [20]. The log-normal shadow model is the most universal of those mentioned above. Commonly analysed components of the radio channel is the mean path loss. In the literature many different approaches for the path-loss modelling in various types of wireless systems can be found. The most common methods used for estimation path-loss exponent are the linear regression with the Least Square Method [21], Multivariate Linear Regression [22] or Generalised Additive Model described in Reference [23]. In the case of a non-linear relationship, a non-linear regressions are used [24]. Statistical methods are also used to estimate path-loss exponent. The Bayesian statistics approach to the estimation of the path-loss exponent are used successfully [25]. Some researchers also use hybrid methods combining several approaches. Many more unique methods for creating predictive models have emerged. These approaches are based on machine learning methods, such as back propagation neural networks, support vector regression, or random forest [26].

For improving localisation, the Bayesian filtering method, along with the Kalman method [27], is often applied in relation to systems based on RSSI signal measurements [28,29,30,31,32,33,34,35]. The particle filter (PF) algorithm has been the subject of many modification and extension proposals. The authors in Reference [28] argue that a particle filter is an accurate Bayesian filtering algorithm that can improve the performance of RSSI-based indoor localisation. In their work, they used modified particle filtering extended by a Kalman filter to reduce the impact of multipath effects and noise on the RSSI. In Reference [29], the authors explain that the existing models of device-free localisation are not accurate enough under certain circumstances. They, therefore, proposed a model based on the variability of RSS. Since the model is highly non-linear, they employed a particle filter-based tracking method. They observed that the proposed model corresponds well with experimental measurements. The authors in Reference [30] describe a particle filter algorithm for distance estimation, using multiple antennas on the receiver’s side and only one transmitter. The distance was predicted as the hidden state of a stochastic system and thus a particle filter was used. Two propagation models were applied for modelling, i.e., a log-normal and a ground reflection one. The use of a particle filter in the Novel Cooperative Localisation Algorithm is proposed in Reference [31]. For maritime search and rescue operations, the authors of this paper demonstrated that the measurement information coming from WSN nodes is inaccurate due to the wave shadow effect. They used the particle filter method to reduce measurement errors. Reference [25] presented a statistical approach in the path-loss model, and the estimation of the model parameters was based on the concepts of Bayesian statistics. Three methods were compared: the grid method, the Metropolis-Hasting method and the Gauss-Newton method. It was shown that the Gauss–Newton algorithm provides satisfactory accuracy and consistency compared with the grid and Metropolis–Hastings methods. It was also revealed that taking the parameter uncertainties into account in the positioning phase improves positioning accuracy compared to the methods in which the path-loss parameters are assumed to be known accurately. Reference [32] presents a method that was based on the uncertainty method and depends on the properties of the radio signal propagation. The authors used the uncertainty factor to estimate the distance. A different approach is presented in Reference [33], which describes the optimisation of the path-loss model based on the use of the optimisation algorithms, such as the firefly algorithm and particle swarm optimisation. The result of the operations performed is an effective correction of the estimated distance values. In another paper [34], the generalised extended interpolation method was applied to define the attenuation model for the parameters of the real environment. The developed model additionally allowed consideration of the variety of the parameters of the equipment. In the case of the log-normal shadow model, most experiments were focused on improving the accuracy of the location. In Reference [35], the authors proposed a two-function path loss model. They defined the rules for determining the parameters for small and larger values of estimated distances.

Optimising RSSI measurements is necessary due to the high noise caused by obstacles and sudden signal fading. Additionally, a change in the environmental parameters (temperature, humidity) can affect the indicator without altering the position of the transmitters [11,36,37]. In most works, the impact of precipitation is usually neglected. This is due to errors in estimating the distance in the case of bad weather conditions. In Reference [36], the influence of precipitation was taken into account, making distance estimation more robust. Here, introducing additional parameters to the model leads to the complexity of calculations, but the model is better suited to reality. Reference [37] presented the process of creating a model with additional parameters, such as the Leaf Area Index and Trunk Diameter at Breast Height. Such additional parameters were related to the model enhancement for the given conditions. In this case, redundancy of the parameters defining a given model can induce complications.

The main novelty of this article results from the introduction of a method of dynamic estimation of the path-loss exponent parameter for outdoor RSSI measurements. In this paper, the path-loss exponent was estimated simultaneously with the estimation of the RSSI values using a Bayesian particle filter. Furthermore, the effect of changing the number of particles on the accuracy of the RSSI and the estimation of the path-loss exponent was also investigated.

This work is organised as follows: Section 2 describes the measurement stand, while Section 3 introduces the particle filtering algorithm used to estimate the of path-loss parameter. Section 4 shows the experimental results that demonstrate the advantages of the proposed models and algorithms. Finally, Section 5 presents the paper’s conclusions.

## 2. Measurement Test Stand

The proposed system for measurements of RSSI values between two WSN nodes consists of radio XBee XB24-Z7WIT-004 modules with XBee 2 mW Series 2 wire antenna modules [38] and a programming platform based on the Esp32 module [39]. In order to establish the wireless connection, two Digi XBee modules were used. In both radios, the firmware was set to the XB24-ZB family. This wireless modules operate in the 2.4 GHz band at 250 kbps baud rate. The parameters of the XBee modules are shown in Table 1.

To perform all measurements, an original program in the C/C++ language was written. This handwritten program allowed a connection to be established between the two XBee modules and has the following functionalities:

- sending packets,
- measurement of packet loss,
- RSSI measurement,
- measurement of temperature and humidity,
- displaying the measured values, and
- saving measurements on the SD card.

**Figure 1:**
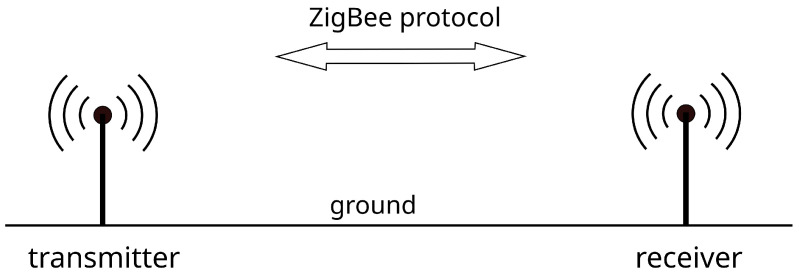
Model of the measuring system.

The distance between the devices was changed and the measurement results were saved on the SD card of one of the modules. After the series of measurements was completed, the data was downloaded and further processed. A schematic diagram of the measurement system is shown in Figure 1. During the measurements, the measuring devices were placed on the platforms at a height of 1 m above the ground. The measurements were made in an open agricultural area.

## 3. Estimation of the Path-Loss Exponent

Modelling signal propagation is an important topic in some applications, such as the Wireless Sensor Network location system. The most typical information used to estimate node distance in a WSN is the Received Signal Strength Indicator [40]. In a open space environment, the power of received signal is determined by applying Friis law (1) and depends on the distance between the transmitter and the receiver [12]:

(1) $$P_{r}\left(d\right)=P_{t}G_{t}G_{r}{\left(\frac{\lambda }{4\pi d}\right)}^{2},$$

where $P_{r}\left(d\right)$ is the signal power, $P_{t}$ is the transmitted signal power, $G_{t}$ is the gain of the transmitter antenna, $G_{r}$ is the gain of the receiver antenna, λ is the wave length, and *d* is the distance.

Moreover, in various environments the power of the transmitted signal decays with distance. In such cases, this relationship can be modelled by the log-normal shadowing model (2), which is widely used in wireless communications:

(2) $$P_{r}\left(d\right)=P_{0}-10n\log\frac{d}{d_{0}}+\xi_{\sigma },$$

where $P_{r}\left(d\right)$ is the received power at distance *d* from the transmitter, $P_{0}$ is the received power measured at reference distance $d_{0}$ from the transmitter, *n* is the path-loss exponent, and $\xi_{\sigma }$ is the zero mean Gaussian noise (the variance of the distribution is $\sigma^{2}$) which represents the random effect caused by shadowing.

The path-loss exponent *n* values typically vary between 1 and 3 in outdoor and 3 to 5 in indoor environments [12,13,35]. However, the path-loss exponent can suddenly change when there are obstacles in sight. Since its value can change with time, a technique to track the exponent is highly desirable. The adoption of a wrong parameter value can induce errors in determining the distance. In such cases, a Bayesian filter is a way to obtain good results.

A particle filter is a Bayesian filter method based on a set of random samples, called particles [41]. Particle (*i*) has an associated weight $w_{i}\left(t\right)$ directly related to likelihood $p(n_{i}\left(t\right)|z\left(t\right))$, where $n_{i}\left(t\right)$ is the state of the *i*-th particle and z(t) is the observed RSSI value, at a given time *t*.

In this paper, the state of the *i*-th particle is composed only by the path-loss exponent parameter *n*. Typically, the Bayesian filtering algorithm has three steps: prediction, update, and resampling. In the prediction step, the random particles *N* are created with an $n_{i}$ value in the range from 0 to 5 with a Gaussian distribution. In the next iteration, the particles update their state randomly. The particle filter used the worst value of standard deviation σ taken from our measurements, e.g., σ = 6.038 m, as a process noise parameter. In the update step, according to the used propagation model shown in (2), we will calculate the following expression:

(3) $$p\left(z\left(t\right)|n_{i}\left(t\right)\right)=\frac{1}{\sigma \sqrt{2\pi }}exp\left(-\frac{{\left(z\left(t\right)-P_{ri}\left(d\right)\right)}^{2}}{2\sigma^{2}}\right),$$

where $P_{ri}\left(d\right)$ is defined by the propagation model (2), but using the appropriate parameter $n_{i}$ stored in the *i*-th particle. Next, the weights are updated and normalised by applying:

(4) $$w_{i}\left(t\right)={\bar{w}}_{i}\left(t-1\right)p\left(z\left(t\right)|n_{i}\left(t\right)\right),$$

and:

(5) $${\bar{w}}_{i}\left(t\right)=\frac{w_{i}\left(t\right)}{\sum_{j=1}^{N}w_{j}\left(t\right)},$$

where ${\bar{w}}_{i}\left(t\right)$ are normalised weights.

To avoid degeneration problems in the particle system, the standard resampling procedure is added [41]. Iterations are then repeated until all experimental points are reached. Finally, the path-loss exponent is computed by means of a weighted sum of the state information from all the particles:

(6) $$n\left(t\right)=\sum_{i=1}^{N}{\bar{w}}_{i}\left(t\right)n_{i}\left(t\right).$$

Next, the procedure is repeated for each measured distances *d*. The implemented particle filter is summarised in Algorithm 1.
**Algorithm 1:** The estimation algorithm of the path-loss exponent.
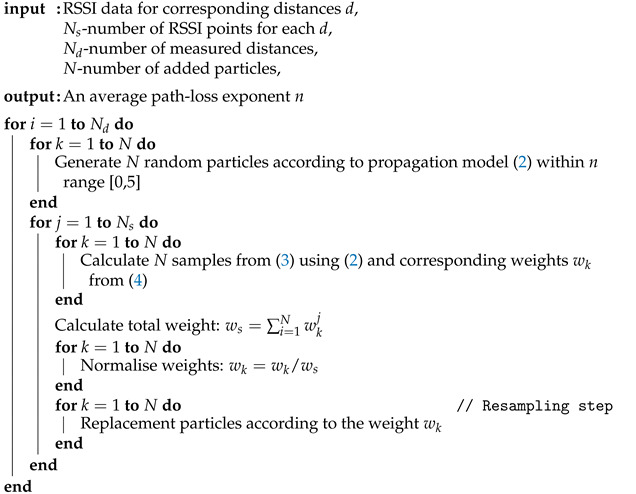


## 4. Results

**Figure 2:**
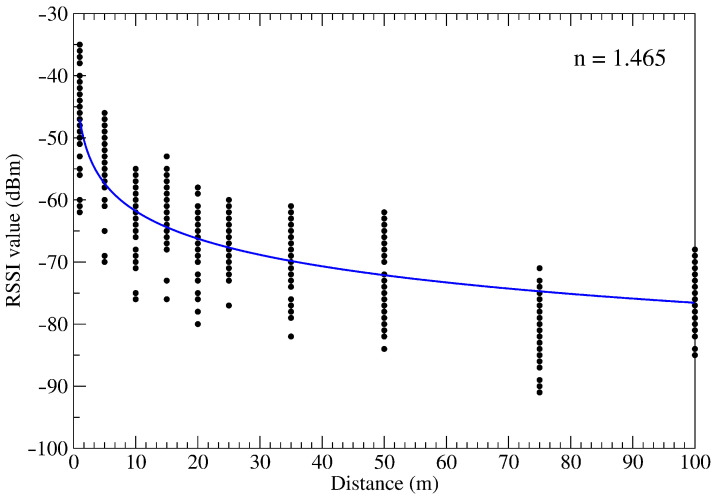
Received signal strength indicator (RSSI) measurements data and calculated path-loss model for different distances. The value of path-loss exponent estimated by approximation is printed in the figure.

Real outdoor RSSI measurements were performed at several distances, ranged from 1 to 100 m between two ZigBee nodes. A series of 60 measurements ($N_{s}$) was carried out for each measured distance (*d*). The results are presented in Figure 2. The average air temperature and humidity during measurements were 16.04 °C and 48.43%. The observed values of the RSSI versus the distance decrease according to the logarithmic function. We approximated RSSI data using a logarithmic function and determined the path-loss exponent *n*. We then calculated *n* using the Scilab computation package and did an approximation of the measurement points. The value obtained in this way was equal to $n_{prx}=1.465$. The results of those calculations are presented in Figure 2 (solid line). This value will be a reference value for the path-loss exponent values $n_{pf}$ estimated later in this paper by using PF.

**Figure 3:**
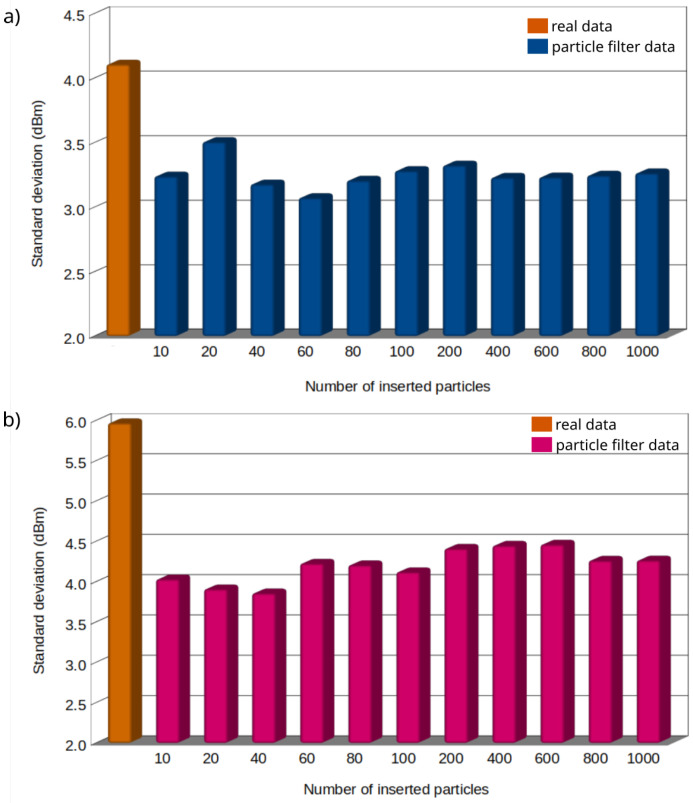
The standard deviation of RSSI data estimated by particle filter for measurement distances equal to 15 m (**a**) and 50 m (**b**) for different numbers of inserted particles. The standard deviation of real experimental data for the same distances are also presented.

The main aim of this work is to measure and denoise the collected RSSI data using a particle filter method. Simultaneously, the path-loss exponent is calculated. In the first stage of the research, the particle filter was applied to the experimental data to test its effectiveness. For this purpose, a series of estimations of RSSI values and their standard deviation were performed. The results of the standard deviation of RSSI estimation for distances equal to 15 m and 50 m and for different numbers of inserted particles are presented in Figure 3. For comparison, in the same Figure 3, the values of the standard deviation of non-estimated measurements (real) are also provided (marked in orange).

The results show that the average statistical error of the estimated values remains at a similar average level of 3.25dBm for *d* = 15 m and 4.20dBm for *d* = 50 m, respectively. Moreover, the values of the standard deviation do not practically depend on the number of inserted particles. Here, its standard deviation shows a much lower value compared to the real measurement results.

**Figure 4:**
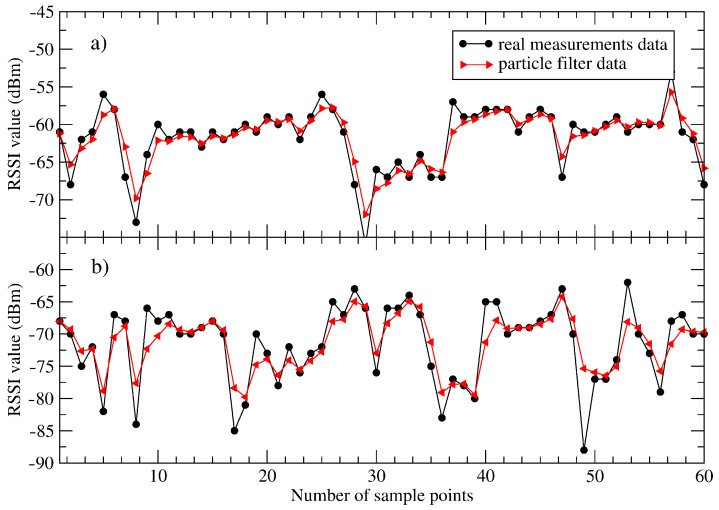
The real RSSI data and estimated particle filter data for the number of inserted particles equal to 100 and distances equal to: 15 m (part **a**) and 50 m (part **b**), respectively. The experimental $N_{s}$ points amount to 60.

Table 2 summarises the values of the standard deviation obtained for the considered values of RSSI measurements. All results were characterised by one relationship. In addition, all estimated RSSI values are characterised by a lower spread of numerical values as compared to the real measurements. Figure 4 reveals that the estimated RSSI values approximate the experimental data very well, resulting in the lowering of the value of the standard deviation.

The above results demonstrate the usefulness of the particle filter for estimating RSSI data. It should be emphasised that RSSI data directly affect the value of the path-loss exponent. Subsequently, we used the algorithm to estimate the RSSI data, as well as the path-loss exponent, i.e., we applied the same algorithm to real measurement data, $P_{r}\left(i\right)$, to estimate $n_{pf}$. As before, the path-loss exponent estimation was performed for a different number of particles. The results are shown in Table 3. The obtained values of $n_{pf}$ differ from the reference value $n_{ax}$ by less than 2%.

**Figure 5:**
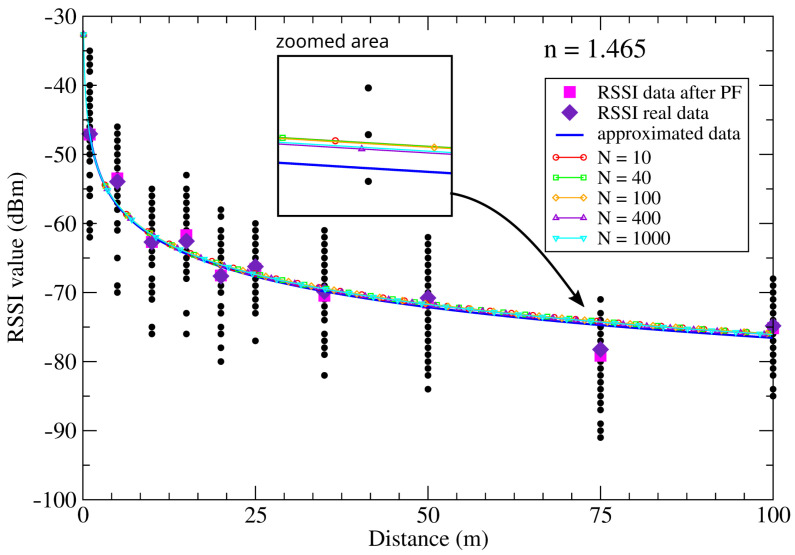
Approximated and estimated path-loss model for different distances. The inset shows the zoomed last point of the presented results.

Figure 5 summarises our results. We can see that the path-loss exponent curves for data approximated and estimated by PF are very similar. Indeed, the difference between the RSSI results for a distance of 75 m does not exceed 0.5dBm (see zoomed part of the figure). For different numbers of inserted particles, differences between the estimated values are even smaller.

Figure 5 also shows the mean values of the raw RSSI measurements (marked with diamond) and the corresponding RSSI values after using a particle filter (marked with squares). The observed mean values are similar to each other. Note that the mean RSSI values after using the filter better define the shape of the approximated curve. This is a direct result of the use of a Bayesian filter which is a non-linear filter [42].

## 5. Conclusions

We successfully used the Bayesian particle filter to estimate the path-loss exponent for experimental RSSI measurements. We realise that comparing the value of the estimated $n_{pf}$ with $n_{ax}$ is not practical. However, it gives us information about the quality of the filter used and confirms that Bayesian particle filters can be successfully used to estimate *n*.

In future work, we intend to investigate the effect of changing the inserted particles on the efficiency of the particle filter. Next, we plan to test the stability of a chosen filter on the gradual reduction in the number of experimental points (input RSSI data). This will be the topic of future research.

The most important results of this study include the following:

- The particle filter can be successfully used to predict the path-loss exponent.
- The standard deviation value of the estimated RSSI data is approximately 30% lower than that of the original data.
- Increasing the number of inserted particles does not significantly raise the quality of the estimated results, finally extending the computation time.

## Figures and Tables

**Table 1:** Summary of XBee module parameters.

Model	XBee XB24-Z7WIT-004
Protocol	ZigBee
Transmission speed	250 kbps
Inner range up to	40 m
Outer range up to	120 m
Frequency	2.4 GHz
Communication	UART
Output power	2 mW
Antenna	Omnidirectional wire

**Table 2:** The standard deviation of the RSSI (dBm) after particle filter estimation for different measurement distances. The last line in the table contains the standard deviation of the RSSI for unchanged experimental data.

N	1 m	5 m	10 m	15 m	20 m	25 m	35 m	50 m	75 m	100 m
10	4.7380	4.9273	3.0805	3.2388	3.8798	3.3774	4.8484	4.0309	3.5413	2.6726
20	4.6047	4.3316	3.2480	3.5055	3.5714	3.3771	4.7689	3.9080	3.7422	2.9418
40	4.7551	4.6782	3.4310	3.1773	3.9372	3.3068	4.8925	3.8574	3.8550	3.3002
60	4.6849	4.6651	3.5001	3.0721	3.6401	3.3065	4.9909	4.2261	3.7606	3.1380
80	4.6858	4.7416	3.4993	3.2059	3.7123	3.3036	5.1008	4.2058	3.7228	3.0494
100	4.5819	4.7945	3.3329	3.2812	3.6523	3.2285	5.0231	4.1202	3.6936	3.0661
200	4.6767	4.9602	3.4595	3.3238	3.7904	3.4360	4.9966	4.4103	3.8727	3.1008
400	4.6504	4.8714	3.3543	3.2291	3.6616	3.4145	5.0875	4.4483	3.8143	3.1369
600	4.6996	4.8601	3.3951	3.2314	3.6337	3.3746	5.0485	4.4613	3.8584	3.1408
800	4.6603	4.7929	3.3253	3.2452	3.7226	3.4023	5.0324	4.2627	3.8850	3.2024
1000	4.6514	4.7766	3.3454	3.2629	3.6548	3.3802	5.0670	4.2654	3.8538	3.1575
Real	6.0211	6.0380	4.4740	4.1058	4.8169	4.2999	5.7238	5.9673	5.0651	4.2553

**Table 3:** The estimated path-loss exponent $n_{pf}$ for different numbers of inserted particles. The approximated path-loss exponent: $n_{prx}=1.465$.

Number of Inserted Particles	Estimated Path-Loss Exponent
10	1.4421
20	1.4472
40	1.4423
60	1.4494
80	1.4522
100	1.4432
200	1.4482
400	1.4497
600	1.4477
800	1.4468
1000	1.4463

## Data Availability

Not applicable.
